# Linear Growth and Fat and Lean Tissue Gain during Childhood: Associations with Cardiometabolic and Cognitive Outcomes in Adolescent Indian Children

**DOI:** 10.1371/journal.pone.0143231

**Published:** 2015-11-17

**Authors:** Ghattu V. Krishnaveni, Sargoor R. Veena, Krishnamachari Srinivasan, Clive Osmond, Caroline H. D. Fall

**Affiliations:** 1 Epidemiology Research Unit, CSI Holdsworth Memorial Hospital, Mysore, India; 2 St. John’s Research Institute, St. John’s National Academy of Health Sciences, Bangalore, India; 3 MRC Lifecourse Epidemiology Unit, University of Southampton, Southampton General Hospital, Southampton, United Kingdom; Sickle Cell Unit, JAMAICA

## Abstract

**Background:**

We aimed to determine how linear growth and fat and lean tissue gain during discrete age periods from birth to adolescence are related to adolescent cardiometabolic risk factors and cognitive ability.

**Methods:**

Adolescents born to mothers with normal glucose tolerance during pregnancy from an Indian birth cohort (N = 486, age 13.5 years) had detailed anthropometry and measurements of body fat (fat%), fasting plasma glucose, insulin and lipid concentrations, blood pressure and cognitive function. Insulin resistance (HOMA-IR) was calculated. These outcomes were examined in relation to birth measurements and statistically independent measures (conditional SD scores) representing linear growth, and fat and lean tissue gain during birth-1, 1–2, 2–5, 5–9.5 and 9.5–13.5 years in 414 of the children with measurements at all these ages.

**Results:**

Birth length and linear growth at all ages were positively associated with current height. Fat gain, particularly during 5–9.5 years was positively associated with fat% at 13.5 years (0.44 SD per SD [99.9% confidence interval: 0.29,0.58]). Greater fat gain during mid-late childhood was associated with higher systolic blood pressure (5–9.5 years: 0.23 SD per SD [0.07,0.40]) and HOMA-IR (5–9.5 years: 0.24 [0.08,0.40], 9.5–13.5 years: 0.22 [0.06,0.38]). Greater infant growth (up to age 2 years) in linear, fat or lean components was unrelated to cardiometabolic risk factors or cognitive function.

**Conclusion:**

This study suggests that factors that increase linear, fat and lean growth in infancy have no adverse cardiometabolic effects in this population. Factors that increase fat gain in mid-late childhood may increase cardiometabolic risk, without any benefit to cognitive abilities.

## Introduction

Recent research has shown that birth weight, and childhood weight gain, are related to adult cardiovascular and metabolic health. Associations of weight at different ages between birth and adulthood with cardiometabolic outcomes have shown opposing directions; for example lower birth weight, but greater childhood weight gain, are consistently associated with a higher risk of adult cardiovascular disease (CVD) and type 2 diabetes (T2DM) [1–3]. Weight gain during infancy (the first 1–2 postnatal years) is less consistently related to these outcomes; while some studies have shown that higher infant weight gain is associated with a lower adult CVD and T2DM risk [1–3], others have shown an increased obesity and cardiometabolic risk [4–5], and others have found no associations [6]. This creates a dilemma for paediatricians in low- and middle-income countries (LMIC) who, given high rates of low birth weight and infant growth faltering, tend to promote early weight gain. This strategy is thought to improve survival and protect neurodevelopment, but may exacerbate later non-communicable disease (NCD) risk [7].

Adair et al. recently showed the importance of distinguishing linear growth from ‘soft tissue’ growth [8]. They used a new 2-way method of conditional growth analysis to examine associations of height growth independent of weight gain, and weight gain independent of height growth (relative weight gain), during discrete age periods, with adult cardiometabolic and human capital outcomes using longitudinal data from five LMIC cohorts (Consortium on Health Oriented Research in Transitional Societies/COHORTS). Greater relative weight gain at all postnatal ages was associated with higher body mass index (BMI), fat mass, fat-free mass, blood pressure (BP) and diabetes risk, but these associations were markedly strong for weight gain after mid-childhood (4–8 years of age). In contrast, linear growth from birth-2 years was positively associated with human capital outcomes such as adult stature and attained schooling, only weakly positively associated with BP, and unrelated to diabetes risk. They concluded that promoting weight gain from mid-childhood onwards may substantially increase adiposity and NCD risk, while promoting linear growth during infancy is likely to benefit human capital without NCD risk. A limitation of their analysis was the inability to separate weight gain into fat and fat-free gain, because the only measurements available were height and weight.

We have now extended their method to a 3-way conditional analysis, using data from an Indian birth cohort, in which children had detailed anthropometry from birth to 13.5 years of age [9]. This enabled an analysis of the independent associations of linear (height) growth, fat (skinfolds) gain, and non-fat weight gain (residual weight) during discrete postnatal age periods with cardiometabolic and cognitive outcomes at 13.5 years. We hypothesised that greater linear growth and non-fat weight gain, especially in infancy, would be associated with lower cardiometabolic risk factors and higher cognitive function score, while greater fat gain at all ages, especially in mid-late childhood, would be associated with greater general and central adiposity and higher cardiometabolic risk factors.

## Methods

### Mysore Parthenon Cohort

During 1997–1998, 830 women attending the antenatal clinics of the Holdsworth Memorial Hospital (HMH), Mysore, India, and matching our eligibility criteria (no known history of diabetes, intention to deliver at HMH, singleton pregnancy) had an oral glucose tolerance test (OGTT) at 30±2 weeks gestation; 785 women completed the OGTT (S1 Fig). Gestational diabetes mellitus (GDM) was diagnosed in 49 women using the Carpenter-Coustan criteria [10]. Six-hundred and sixty-three of these women delivered live babies without major congenital anomalies at HMH. Neonatal anthropometry, including weight, crown-heel length, and subscapular and triceps skinfold thickness, was performed within 72-hours of birth as described before [11]. On follow-up, 25 children died, and 8 were excluded because of major medical conditions. Anthropometry, including measurements of height, weight and subscapular and triceps skinfold thickness was performed in the remaining children annually until 5 years of age, and 6-monthly thereafter. Fat and fat-free mass were measured using bioimpedance from 5-year onwards.

Anthropometric measurements were standardized by regular inter- and intra-observer variation studies. The inter-observer co-efficient of variation (CV) was <1% for weight and crown-heel length/ height at birth and subsequent follow-up points. The inter-observer CV for triceps skinfold thickness was 2.1% at birth, 0.4% during infancy and ≤6% during 5 and 9.5 year follow-ups. For subscapular skinfold thickness, CV ranged from 3.7% at birth and infancy to 8.2% at 5 year follow-up.

At 13.5 years, 545 children were available for follow-up. Detailed anthropometry was performed, including the measurements of weight (Salter, Tonbridge, Kent, UK), height (Microtoise, CMS instruments, UK), and triceps and subscapular skinfold thickness measurements (Harpenden callipers, CMS instruments). The inter-observer CV was <1% for height and weight, 1.3% for triceps and 4.5% for subscapular skinfold thickness. Percentage body fat (fat%) was measured using bioimpedance (Bodystat, Quadscan 4000, Isle of Man, UK). This method has been shown to give reliable estimates of fat and fat-free mass [12,13]. We have previously observed in a subgroup of the present cohort that bioimpedance method was useful for measuring group-level changes in body fat [14].

Systolic and diastolic blood pressure (BP) were measured using an automated BP monitor (Dinamap 8100, Criticon, FL, USA). Waist-to-hip ratio (WHR) was calculated from waist and hip circumferences as a measure of central adiposity. Pubertal growth was assessed as the stage of breast development in girls and genital development in boys using Tanner’s method [15]. The socio-economic status (SES) of the family was determined using the Standard of Living Index designed by the National Family Health Survey-2 [16]. Plasma glucose, insulin and lipid concentrations were measured using fasting blood samples. Assays were carried out at the Diabetes Unit, KEM Hospital, Pune, India, whose laboratory is a member of the UK National External Quality Assessment Service (NEQAS) quality control programme for insulin assays. Glucose and lipid concentrations were measured by standard enzymatic methods (Hitachi-902, Roche, Germany). Insulin was measured using the enzyme-linked immunosorbent assay (Mercodia Ultrasensitive, Mercodia AB, Uppsala, Sweden). Inter- and intra-assay CV was <7.0%. Insulin resistance was estimated using Homeostasis Model Assessment for insulin resistance (HOMA-IR) [17].

Cognitive function was assessed using tests from the Kaufman Assessment Battery for children-second edition, 2004 and additional tests as described before [18]. These tests had been adapted and validated to ensure their applicability in the local cultural context [19]. The cognitive domains tested were short-term memory (Word order, Coding), long-term memory and retrieval ability (Atlantis), visuo-spatial ability (Koh’s block design), reasoning (Pattern reasoning) and language production (Verbal fluency). All tests were administered to each child in a single session of 60–90 min at the hospital research centre, in a separate quiet room by one of 3 trained masters’ level child psychologists in the local Kannada language. The inter-observer CV was <1% for all the tests except verbal fluency (1.8%). In addition to the individual test scores, a mean cognitive score as the average of all the individual scores was calculated.

The Holdsworth Memorial Hospital ethics review committee approved the study, and informed written consent was obtained from the parents and assent from children.

### Statistical methods

#### Assessment of size and growth

**Fig 1:**
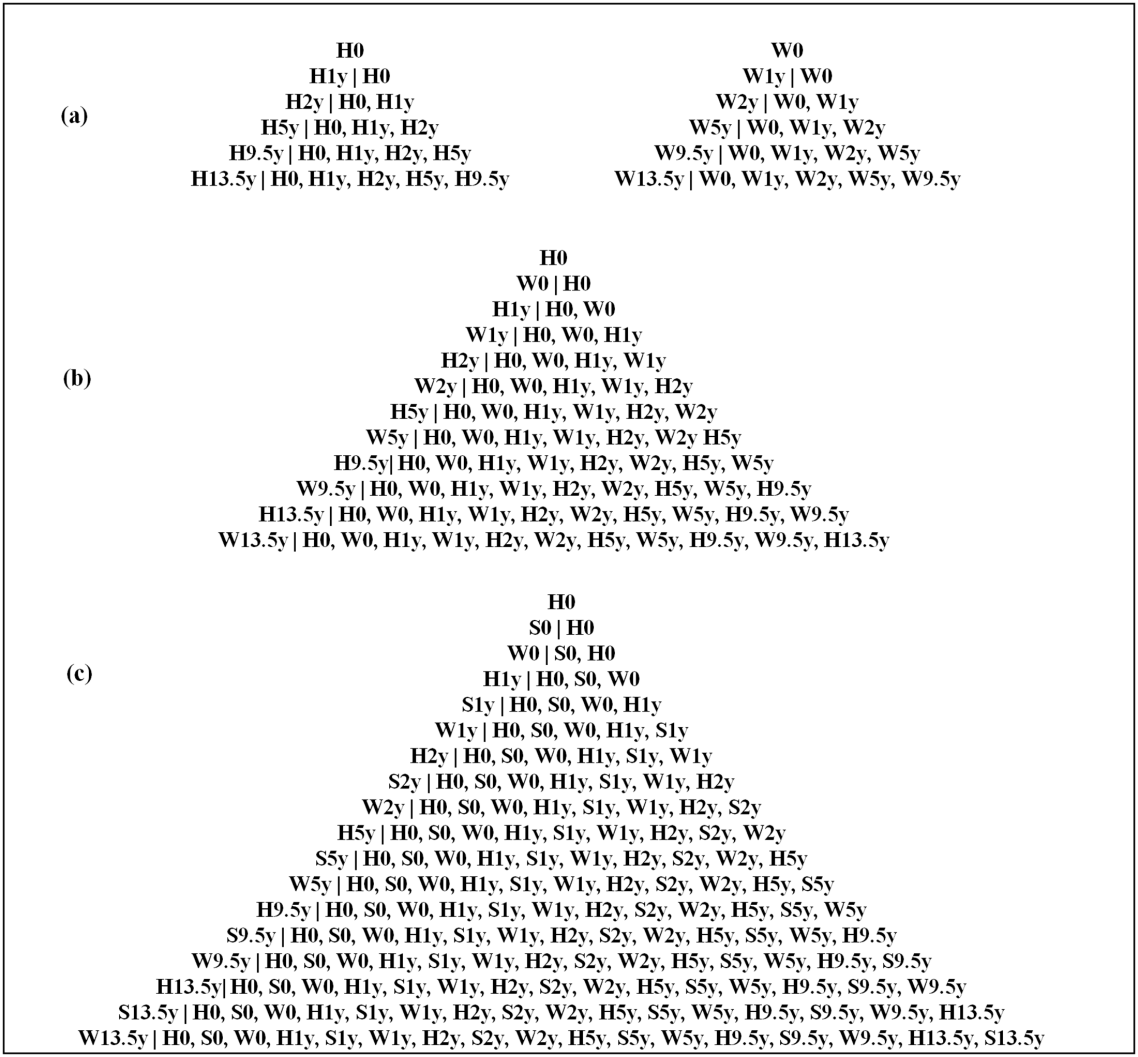
Schematic Diagram to Show the Commonly Used and Extended Methods of Deriving Conditional Growth Variables. H: height/length; S: sum of skinfold thickness; W: weight. Each pyramid shows the calculation of a set of conditional variables; (a) shows the use of a single measurement eg. weight or height; (b) shows two measurements combined (weight and height); and (c) shows three measurements as used in our analysis (height, skinfolds and weight). Within each pyramid, each row shows the calculation of one conditional variable, from length at birth at the top to (at the base) 13.5 year conditional weight or height (a), conditional relative weight (b) or conditional lean tissue gain (c). These variables are measures of the residuals from regression models in which the measure to the left of the vertical bar is the outcome, and measures to the right of the vertical bar are the predictors. (a): In the first method, current weight or height was conditioned respectively on all previous measurements of the same type. (b): In the second method described by Adair et al (8), ‘linear growth’ was current height conditioned on all previous height and weight measurements; ‘relative weight gain’ was current weight conditioned on current height and all previous weight and height measures. (c): In our extended method, ‘linear growth’ was current height conditioned on all previous height, skinfold and weight measurements; ‘fat gain’ was current skinfold thickness conditioned on current height and all previous height, skinfold and weight measures; ‘lean tissue gain’ was current weight conditioned on current height and skinfolds, and all previous height, skinfold and weight measures.

Within an individual, different body measurements at a given age, and the same body measurement at different ages, are strongly correlated. This makes it difficult to assess the relationship of the growth of specific tissues during specific age periods to later outcomes. Conditional growth analysis, in which standardised residuals are derived from regressing current size on all prior size measurements, is one way of overcoming this (Fig 1a) [20]. It has been most commonly applied to single body measurements such as weight or height (Fig 1a) [21]. Adair and colleagues developed the method further by combining two measurements (weight and height; Fig 1b) [8]. Conditional height (representing linear growth independent of weight gain) was current height accounting for all prior height and weight measures but not current weight, and weight gain independent of linear growth was current weight accounting for current height and all prior weight and height measures. In the current analysis we combine three measurements (height, sum of skinfold thickness and weight) (Fig 1c). We used skinfold thickness measurements rather than fat mass measured using bioimpedance method as this was not measured serially from birth. Conditional length/height (representing ‘*linear growth’*) is current length/height accounting for all prior length/height, skinfold and weight measurements but not current skinfolds and weight; conditional fat (representing ‘*fat gain’*) is current skinfold thickness accounting for all prior length/height, skinfolds and weight, and current height but not current weight; and conditional lean (representing ‘*lean tissue gain’*) is current weight accounting for all prior length/height, skinfolds and weight and current height and skinfolds.

The conditional variables measure the difference between the size attained and that expected from the child’s earlier size measurements and selected current measurements, using data from the whole cohort. A positive value indicates greater (or faster) than expected growth, and a negative value indicates less (or slower) growth than expected. An advantage of this method is that, by construction, the conditional variables at all ages for any individual are uncorrelated with each other, and can be included together in a regression model. A disadvantage is that conditional variables can only be used for those who have valid measurements at every age chosen. In the current study, birth, 1, 2, 5, 9.5 and 13.5 years were selected, to represent growth in early-infancy (birth-1 year), late-infancy (1–2 years), early-childhood (2–5 years), mid-childhood (5–9.5 years) and late-childhood (9.5–13.5 years). We excluded offspring born to diabetic mothers and those whose mothers’ GDM status was unknown. Before deriving the conditional variables, size measurements at the selected ages were converted into SD scores, separately for boys and girls, adjusting for exact age at measurement, using Fisher-Yates’ transformation. The conditional variables themselves (residuals) are also expressed as SD scores. Conditional height, fat and lean variables were derived for all available children, separately for boys and girls, at each age interval.

#### Examining the association between conditional growth and outcomes at 13.5 years

At 13.5 years, 486 children born to normal glucose tolerant mothers were available for follow-up. The analysis was restricted to 414 children who had all the required growth measurements at all ages (85% of eligible children followed up at 13.5 years). Outcome variables (anthropometry, body composition, cardiometabolic risk factors and cognitive function) at 13.5 years were converted into SD scores using Fisher-Yates’ transformation, to enable comparison of effects across outcomes. Linear regression analysis was used to examine associations of conditional height, fat and lean with outcomes, adjusted for age, sex, SES and pubertal stage. Due to a high degree of correlation between growth variables and current anthropometry, we did not additionally adjust for current size or fat measurement in these models. Growth variables for the 9.5–13.5 year interval were not included in the models for anthropometry and body composition outcomes as, by construction, these variables also included relevant 13.5 year measurements. Effect sizes with 99.9% confidence intervals (CI), equivalent to a P-value of <0.001, are presented, rather than 95% CI (P<0.05), to account for multiple testing. Data were analysed using SPSS v 21 (IBM Corp, NY, USA).

## Results

Table 1 describes the general characteristics of the children included for the current analysis. Included children tended to have higher skinfold thickness measurements at 2- and 5-year follow-up rounds compared to those not studied (S1 Table). There was no difference between the studied and not studied children in other growth variables at different age points, or outcomes at 13.5 years.

**Table 1:** Characteristics of the Children at Birth, and at 1, 2, 5, 9.5 and 13.5 Years of Age.

	N	Boys (N = 208)	Girls (N = 206)
**Birth**			
Gestation (weeks)	414	39.0 (1.7)	39.2 (1.6)
Crown-heel length (cm)	414	48.9 (2.3)	48.3 (2.1)
Sum of skinfold thickness (mm)	414	8.3 (1.6)	8.1 (1.6)
Weight (kg)	414	2.906 (0.465)	2.803 (0.388)
**One year**			
Age (yr)	414	1.00 (0.03)	1.00 (0.03)
Crown-heel length (cm)	414	74.0 (2.7)	72.2 (2.7)
Sum of skinfold thickness (mm)	414	14.5 (2.7)	14.4 (2.9)
Weight (kg)	414	8.8 (1.0)	8.0 (1.0)
**Two years**			
Age (yr)	414	2.00 (0.03)	2.00 (0.02)
Crown-heel length (cm)	414	84.4 (3.2)	82.6 (3.1)
Sum of skinfold thickness (mm)	414	14.5 (2.7)	14.4 (2.9)
Weight (kg)	414	10.8 (1.2)	10.2(1.3)
**Five years**			
Age (yr)	414	5.00 (0.04)	5.00 (0.03)
Height (cm)	414	106.3 (4.3)	104.9 (4.3)
Sum of skinfold thickness (mm)	414	12.9 (2.6)	15.1 (3.6)
Weight (kg)	414	15.4 (2.0)	14.9 (2.0)
**9.5 years**			
Age (yr)	414	9.35 (0.10)	9.36 (0.11)
Height (cm)	414	131.3 (5.5)	130.1 (5.9)
Sum of skinfold thickness (mm)	414	15.8 (5.6)	19.6 (6.0)
Weight (kg)	414	25.2 (4.3)	24.7 (4.4)
**13.5 years**			
Age (yr)	414	13.53 (0.14)	13.53 (0.14)
SES (score)	414	39.1 (6.8)	38.2 (7.2)
Pubertal stage (N): ≤2/3/≥4^a^	404	8/44/148 (4/22/74)	40/109/55 (20/53/27)
Height (cm)	414	154.5 (7.7)	152.9 (5.7)
Sum of skinfold thickness (mm)	414	23.0 (12.7)	30.0 (10.3)
Weight (kg)	414	40.7 (8.7)	42.7 (8.0)
Body Fat (%)	414	17.2 (6.6)	25.6 (5.8)
Systolic BP (mmHg)	414	111.2 (8.3)	107.3 (7.8)
Diastolic BP (mmHg)	414	63.0 (6.8)	59.3 (6.9)
Glucose 0 (mmol/l)	409	5.0 (0.5)	5.0 (0.5)
Insulin 0 (pmol/l) ^b^	409	33.4 (21.6,46.7)	44.5 (34.8,58.3)
HOMA-IR ^b^	409	1.2 (0.8,1.8)	1.7 (1.3,2.1)
Total cholesterol (mmol/l)	409	3.5 (0.7)	3.5 (0.7)
Triglycerides (mmol/l)	409	0.8 (0.5)	0.9 (0.4)
HDL-Cholesterol (mmol/l)	409	1.09 (0.27)	1.04 (0.23)
Koh’s Block Design (score) ^c^	414	84.4 (26.7)	82.7 (26.0)
Verbal Fluency (score) ^c^	414	19.8 (4.8)	22.9 (6.1)
Atlantis (score) ^c^	414	79.8 (14.6)	80.7 (13.9)
Pattern Reasoning (score) ^c^	414	14.8 (6.6)	16.3 (6.7)
Word Order (score) ^c^	414	18.5 (3.5)	19.2 (3.9)
Coding (score) ^c^	414	44.2 (10.0)	50.4 (11.3)
Mean cognitive score (score)	414	43.6 (7.7)	45.4 (7.6)

SES: Socio-economic status; BP: Blood pressure; HOMA-IR: Homeostasis Model Assessment insulin resistance. Values given are mean (SD), Pubertal stage represents breast development stage in girls and genital development stage in boys; Sum of skinfold thickness is the sum of triceps and subscapular skinfold thickness.

^a^ N (%) or

^b^ median (IQR).

^c^ The cognitive domains tested were visuo-spatial ability (Koh’s block design), language production (Verbal fluency), long-term memory and retrieval ability (Atlantis), reasoning (Pattern reasoning) and short-term memory (Word order, Coding)

Girls who had faster linear growth (0.33 SD per SD [99.9% CI: 0.13,0.53]) and greater lean tissue gain from 5–9.5 years (0.20 SD per SD [0.01,0.40]) were at a more advanced breast development stage; there was no association between conditional growth and pubertal development in boys.

### Conditional growth and height and body composition at 13.5 years

**Fig 2:**
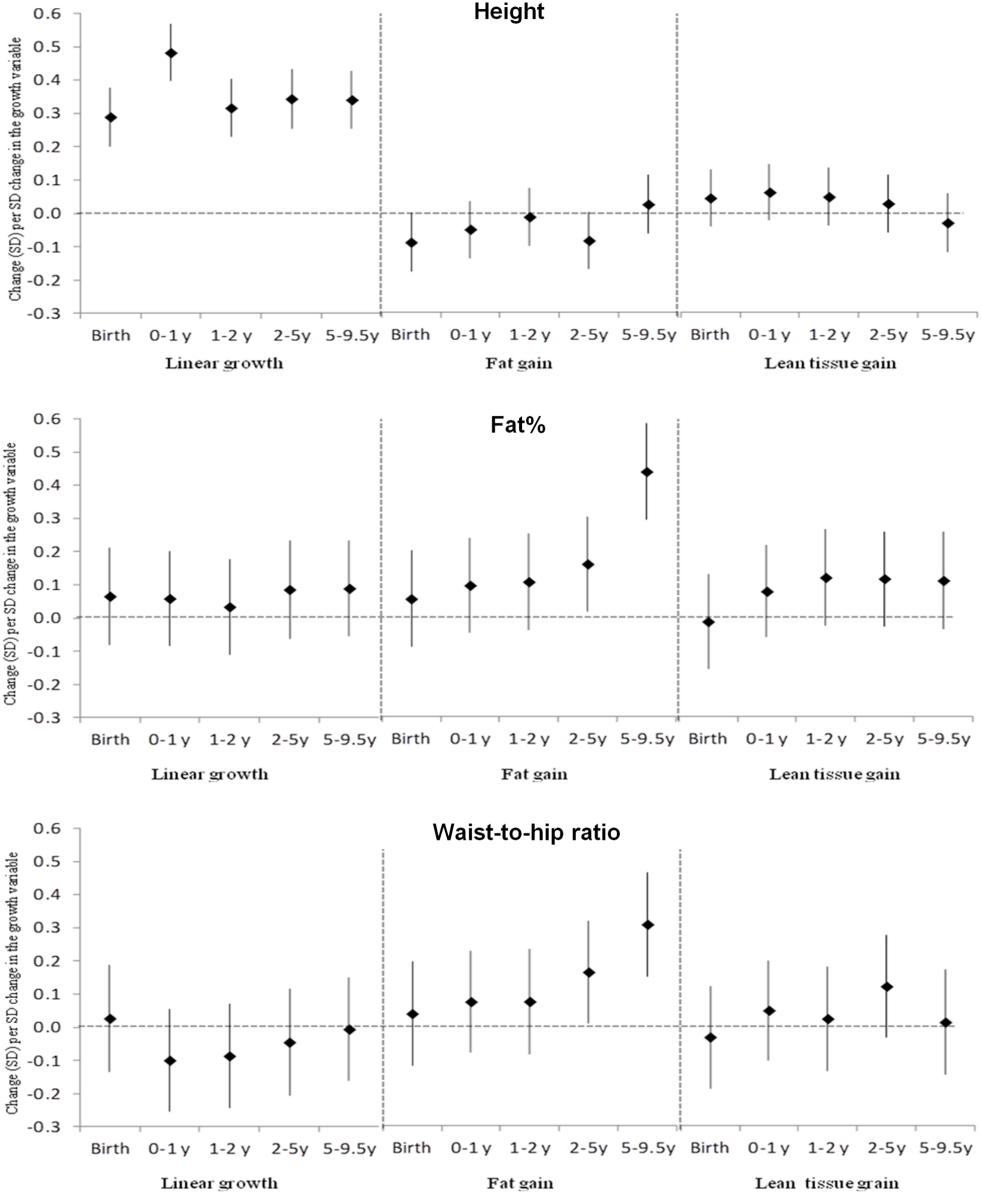
Associations of Linear Growth and Fat and Lean Tissue Gain with Height, Fat% and Waist-to-Hip Ratio at 13.5 Years. Values represent standard deviation/SD (99.9% confidence interval) change in outcome per SD change in growth variable.

Length at birth and linear growth at all age intervals from birth-9.5 years were strongly positively associated with current height (Fig 2, S2 Table). Faster linear growth, especially from 5–9.5 years was associated with higher BMI and sum of skinfold thickness; but there were no associations with WHR and fat% from bioimpedance. More fat at birth was associated with shorter current height. Fat at birth, and fat gain during infancy and childhood were positively associated with current BMI and skinfold thickness. Only fat gain during childhood was positively associated with WHR and fat%. Greater lean tissue gain at all ages after birth was associated with higher 13.5-year BMI. Greater lean tissue gain from 2–5 years was also associated with larger skinfold thickness.

### Conditional growth and cardiometabolic and cognitive outcomes at 13.5 years

**Fig 3:**
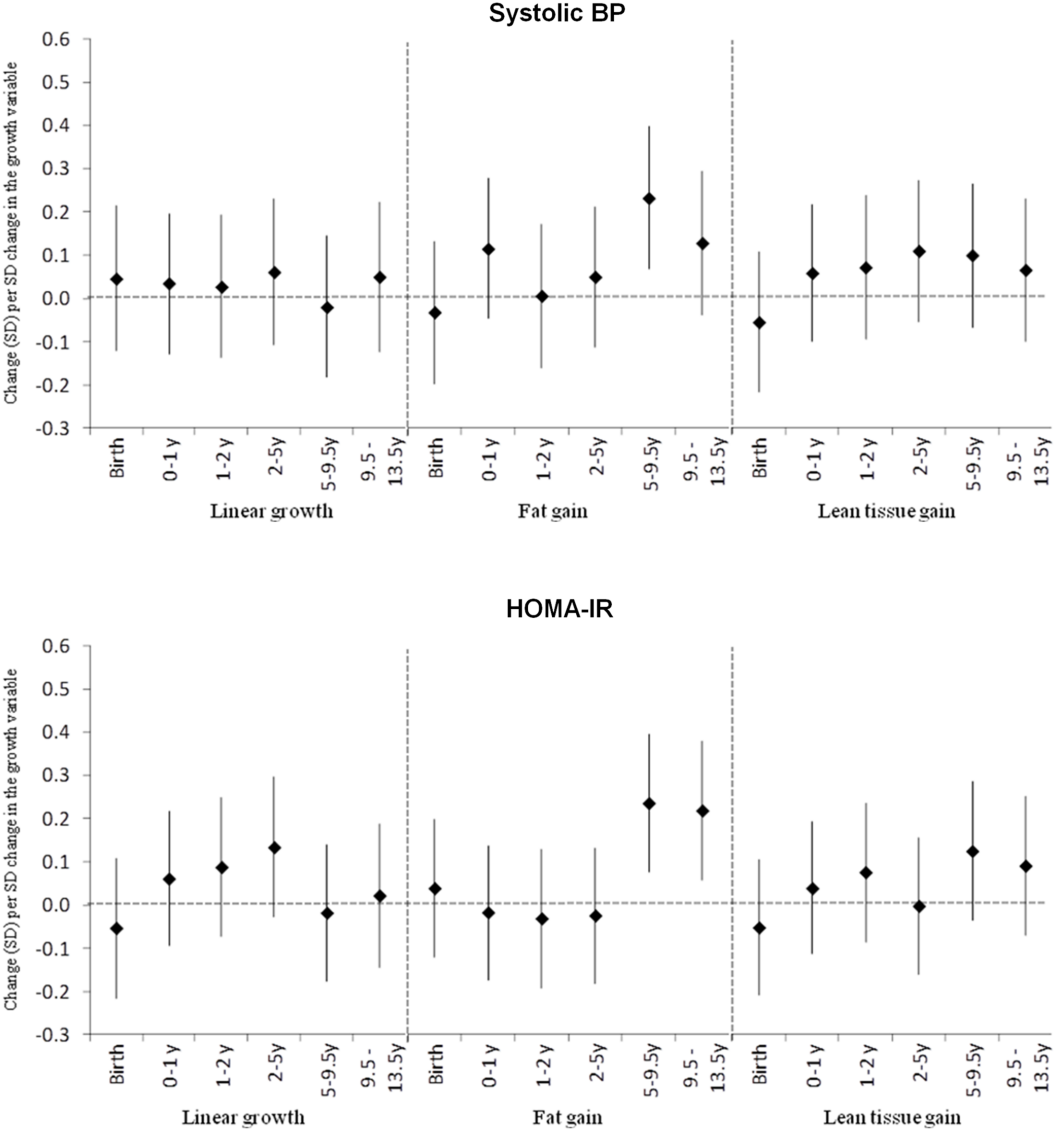
Associations of Conditional Linear Growth and Fat and Lean Tissue Gain with Systolic Blood Pressure (BP) and HOMA-IR. Values represent standard deviation/SD (99.9% confidence interval) change in outcome per SD change in growth variable.

There were no associations of length or lean tissue at birth, or of linear or lean tissue growth at any age with current cardiometabolic risk outcomes (Fig 3, S3 Table). Fat gain after 5 years was positively associated with systolic BP, fasting insulin concentrations and HOMA-IR.

There were no associations between linear growth, fat gain or lean tissue gain at any age interval and current cognitive function (S4 Table).

There were no interactions between sex and growth parameters in relation to the outcomes reported above.

## Discussion

This study exploring associations of linear growth, fat gain and lean tissue gain during different age intervals in infancy and childhood with health-related outcomes at 13.5 years of age showed that faster fat gain in mid-late childhood predicts greater fat% and central adiposity, and higher systolic BP and insulin resistance (HOMA-IR). Although greater neonatal fat and greater fat gain during infancy were associated with greater BMI and skinfolds, they were not associated with cardiometabolic risk factors. Contrary to our hypothesis, there was no evidence for associations of faster linear growth and greater lean tissue gain during early life with a more favourable cardiometabolic or cognitive outcomes.

Major strengths of the study were the availability of detailed neonatal and postnatal anthropometry, including skinfold thickness measurements, and the assessment of risk factors in a large group of healthy adolescents. Though previous studies have examined the independent effects of linear growth and relative weight gain on later cardiovascular and human capital measures, this is the first study to use a 3-way conditional analysis to separate the effects of lean and fat gain from relative weight gain in childhood. We examined associations of growth in children without intra-uterine exposure to hyperglycemia, which is known to influence body size and risk outcomes [22]. A limitation was that about 15% of the children who were followed-up at 13.5 years were not included because they did not have growth measurements available at all age intervals. This could have introduced selection bias, though birth and postnatal anthropometry, and outcome measurements were generally similar in the included and excluded children. Our longitudinal measures of body composition based on anthropometry could be considered rather a crude method of assessing fat and lean mass. Methods such as bioimpedence, dual X-ray absorptiometry (DXA) or doubly-labelled water studies may be more precise. Though bioimpedance was used to measure body fat in later follow-up years in our study, unavailability of these measurements at birth and during infancy precluded their use in the construction of growth variables. However, to our knowledge, no cohorts have used these sophisticated methods at frequent intervals throughout infancy and childhood. Bioimpedence and DXA are difficult to perform in infants, serial DXA measurements would be precluded in children due to radiation exposure, and doubly-labelled water would be time-consuming [23].

Growth during different stages of the lifecourse has been thought to influence later health outcomes differently, though observations have not been consistent. In New Delhi, India, young adults who developed T2DM and glucose intolerance had a lower BMI during infancy, but had accelerated childhood weight or BMI gain [3]. In Pune, greater gain in all measurements after six months of age was associated with higher CVD risk markers at six years [24]. In the Avon Longitudinal Study of Parents and Children in the UK, weight and height gain both during infancy and childhood predicted higher systolic BP at 10 years; the association was strongest for post-infancy weight gain [25]. In the COHORTS data, weight gain and/or linear growth during infancy was associated with increased fat-free mass [26], taller adult height [27] and more attained schooling [28]. Most of these studies assessed growth using either weight or height gain. Therefore, due to strong correlations between different body measurements, independent effects of components of size are not clear. Adair et al. attempted to overcome this issue by disentangling the effects of linear growth and relative weight gain, and showed that rapid linear growth during the first two years of life was associated with increased adult height and schooling [8]. Higher relative weight gain in mid-late childhood was associated with an increased risk of adult overweight, elevated BP and diabetes.

Our extended conditional method further elucidates the independent influences of linear growth, fat gain and lean tissue gain during infancy and childhood on later outcomes. Children who had a greater gain in the fat component of relative weight gain, particularly during mid-late childhood, developed higher cardiometabolic risk markers. There were no obvious adverse or beneficial associations of faster infant linear, fat or lean tissue growth with cardiometabolic and cognitive outcomes. It is encouraging that later linear and lean tissue growth did not seem to predict higher cardiometabolic risk. This may have important implications for improving quality of life through promoting growth in these components in those who had compromised early growth, because linear and lean tissue gain even in childhood is important for optimum adulthood body composition [29]. Greater length at birth and faster linear growth at all ages were related to increased current height as expected. Interestingly, greater neonatal fat was related to shorter current height. This may reflect the fat-sparing effect described in growth-retarded Indian babies (thin-fat phenotype) [30], who are more likely to be stunted in later childhood [31]. As paternal height is an important predictor of the skeletal and fat-free tissue growth in the fetus [32], offspring born to short fathers, who are likely to attain shorter final height themselves, may have increased relative adiposity at birth.

Notably, linear and lean growth during infancy was not related to cognitive performance in our study. Considering the importance of early growth for optimum neuro-cognitive development, we expected a strong association of infant growth on cognitive function. However, our finding is consistent with a recent systematic review which showed a weak positive or no association of weight or height gain during infancy on subsequent cognitive function indices in non-small-for-gestational-age children [33]. It has been suggested that adverse effects of early nutritional deficiencies on cognitive function can be ‘corrected’ through appropriate environmental and social stimuli even at a later developmental stage [34]. This may be a reason for an absent relationship in our relatively homogenous sample of urban children, for whom the opportunities for learning and environmental interactions are largely similar.

## Conclusions

Previous studies have consistently shown that accelerated weight gain during late childhood predicts greater adult NCD risk; our study suggests that this may correspond to increased fat gain. The long-term implication of growth during infancy and early childhood needs further study. While our findings suggest that early growth up to the age 5 years may have no subsequent adverse cardiometabolic implications, some studies, mainly from high-income settings, have observed greater NCD risk in association with rapid infant weight gain. Similar analyses to ours, or using more advanced serial body composition measurements through infancy and childhood, in other populations, would help to clarify this issue. This is important for public health strategies in countries like India where high rates of fetal growth retardation may lead to an increased prevalence of childhood morbidity and mortality. In these settings, infancy provides an important and effective window of opportunity to maximise survival by promoting faster growth. Well-designed intervention studies may provide more conclusive evidence regarding the benefits and risks of promoting early growth.

## Supporting Information

S1 Fig Flow Chart to Illustrate the Rate of Participation and Attrition at Major Follow-up Stages of the Parthenon Study. HMH: Holdsworth Memorial Hospital; OGTT: Oral glucose tolerance test; GDM: Gestational diabetes mellitus
(TIF)

S1 Table Characteristics of the Included and Excluded Children at Birth, and at 1, 2, 5, 9.5 and 13.5 Years of Age. ^a^ Median (IQR). β (99.9% CI) values represent the difference (included-excluded) between children who were included and not included for the conditional analysis; values derived using linear regression analysis, adjusted for age, sex, and (for 13.5 year outcomes), socio-economic status and pubertal stage. For gestational age and age variables: values adjusted for sex. BP: Blood pressure; HOMA-IR: Homeostasis Model Assessment insulin resistance; Sum of skinfold thickness: sum of triceps and subscapular skinfold thickness.
(DOC)

S2 Table Regression Coefficients for Associations Between Conditional Growth Variables and Anthropometry and Body Composition at 13.5 Years. β represents SD change in outcome variables per SD change in growth over that expected at a given age. Analyses adjusted for age, sex, socio-economic status and pubertal stage. SD: standard deviation, CI: confidence interval; Sum of skinfold thickness: sum of triceps and subscapular skinfold thickness.
(DOC)

S3 Table Regression Coefficients for Associations between Conditional Growth Variables and Cardiometabolic Risk Factors at 13.5 Years. β represents SD change in outcome variables per SD change in growth over that expected at a given age. Analyses adjusted for age, sex, socio-economic status and pubertal stage. SD: standard deviation, CI: confidence interval; BP: blood pressure; HOMA-IR: Homeostasis Model Assessment insulin resistance.
(DOC)

S4 Table Regression Coefficients for Associations between Conditional Growth Variables and Cognitive Outcomes at 13.5 Years. β represents SD change in outcome variables per SD change in growth over that expected at a given age. Analyses adjusted for age, sex, socio-economic status and pubertal stage. SD: standard deviation, CI: confidence interval.
(DOC)
