# Supplementary figures and images for: Linear Growth and Fat and Lean Tissue Gain during Childhood: Associations with Cardiometabolic and Cognitive Outcomes in Adolescent Indian Children
Source: PLoS One. 2015 Nov 17;10(11):e0143231. doi: 10.1371/journal.pone.0143231 (PMC4648488; doi:10.1371/journal.pone.0143231)

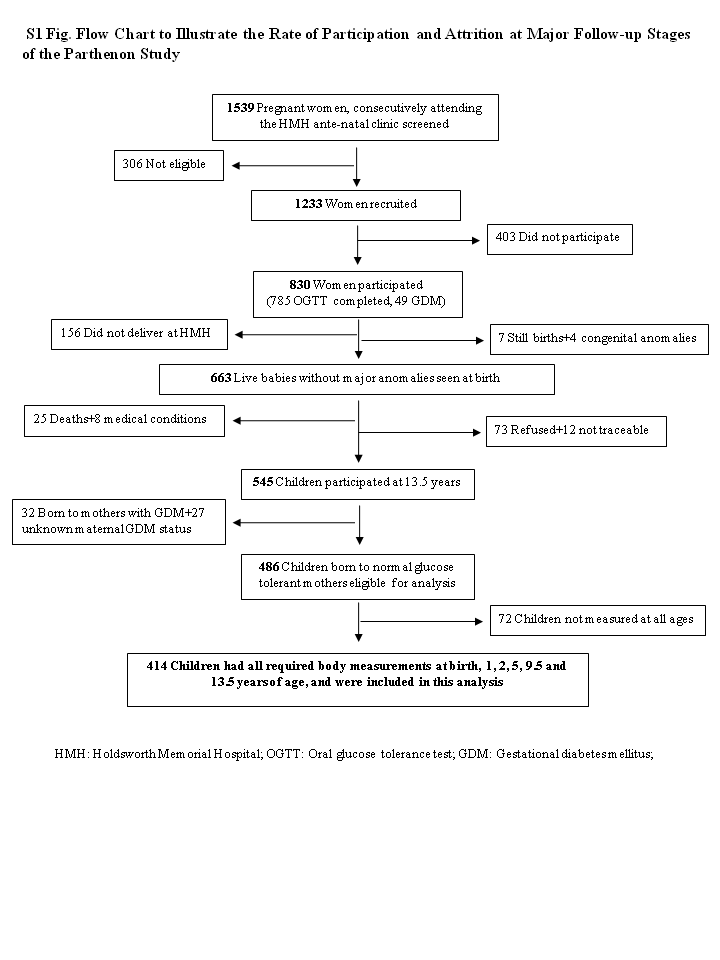

Supplement: S1 Fig — HMH: Holdsworth Memorial Hospital; OGTT: Oral glucose tolerance test; GDM: Gestational diabetes mellitus (TIF) [file pone.0143231.s001.tif]
