# Supplementary material for: Linear Growth and Fat and Lean Tissue Gain during Childhood: Associations with Cardiometabolic and Cognitive Outcomes in Adolescent Indian Children
Source: PLoS One. 2015 Nov 17;10(11):e0143231. doi: 10.1371/journal.pone.0143231 (PMC4648488; doi:10.1371/journal.pone.0143231)
Supplement: S1 Table — a Median (IQR). β (99.9% CI) values represent the difference (included-excluded) between children who were included and not included for the conditional analysis; values derived using linear regression analysis, adjusted for age, sex, and (for 13.5 year outcomes), socio-economic status and pubertal stage. For gestational age and age variables: values adjusted for sex. BP: Blood pressure; HOMA-IR: Homeostasis Model Assessment insulin resistance; Sum of skinfold thickness: sum of triceps and subscapular skinfold thickness. (DOC) [file pone.0143231.s002.doc]

**S1 Table. Characteristics of the Included and Excluded Children at Birth, and at 1, 2, 5, 9.5 and 13.5 Years of A**ge.

|  | **Included** | | | **Excluded** | | |  |  | |  |
| --- | --- | --- | --- | --- | --- | --- | --- | --- | --- | --- |
|  | **N** | **Mean (SD)** | | **N** | **Mean (SD)** | | **β** | **99.9% CI** | | **P** |
| **Birth** |  |  | |  |  | |  |  | |  |
| Gestation (weeks) | 414 | 39.1 | (1.7) | 72 | 39.0 | (1.8) | 0.11 | -0.60 | 0.82 | 0.6 |
| Crown-heel length (cm) | 414 | 48.6 | (2.2) | 70 | 48.4 | (2.4) | 0.13 | -0.72 | 0.99 | 0.6 |
| Sum of skinfold thickness (mm)* | 414 | 8.5 | (1.6) | 70 | 8.4 | (1.8) | 0.18 | -0.49 | 0.86 | 0.4 |
| Weight (kg) | 414 | 2.856 | (0.432) | 72 | 2.8013 | (0.467) | 0.04 | -0.13 | 0.21 | 0.5 |
| **One year** |  |  |  |  |  |  |  |  |  |  |
| Age (yr) | 414 | 1.00 | (0.03) | 32 | 1.01 | (0.04) | -0.01 | -0.03 | 0.01 | 0.03 |
| Crown-heel length (cm) | 414 | 73.1 | (2.8) | 32 | 73.3 | (2.5) | -0.46 | -2.09 | 1.17 | 0.3 |
| Sum of skinfold thickness (mm) | 414 | 14.4 | (2.8) | 32 | 13.8 | (3.4) | 0.60 | -1.13 | 2.33 | 0.2 |
| Weight (kg) | 414 | 8.4 | (1.1) | 32 | 8.4 | (1.0) | -0.09 | -0.71 | 0.54 | 0.6 |
| **Two years** |  |  |  |  |  |  |  |  |  |  |
| Age (yr) | 414 | 2.00 | (0.03) | 48 | 2.00 | (0.03) | -0.01 | -0.02 | 0.01 | 0.09 |
| Crown-heel length (cm) | 414 | 83.5 | (3.3) | 48 | 83.8 | (3.3) | -0.32 | -1.91 | 1.28 | 0.5 |
| Sum of skinfold thickness (mm) | 414 | 14.7 | (3.0) | 48 | 13.9 | (2.7) | 0.90 | -0.61 | 2.40 | 0.05 |
| Weight (kg) | 414 | 10.5 | (1.2) | 48 | 10.3 | (1.1) | 0.12 | -0.49 | 0.72 | 0.5 |
| **Five years** |  |  |  |  |  |  |  |  |  |  |
| Age (yr) | 414 | 5.00 | (0.0) | 61 | 5.01 | (0.06) | -0.01 | -0.02 | 0.01 | 0.4 |
| Height (cm) | 414 | 105.6 | (4.3) | 61 | 105.4 | (4.2) | 0.15 | -1.80 | 2.09 | 0.8 |
| Sum of skinfold thickness (mm) | 414 | 14.0 | (3.3) | 61 | 13.2 | (3.1) | 0.90 | -0.55 | 2.34 | 0.04 |
| Weight (kg) | 414 | 15.2 | (2.0) | 61 | 14.8 | (1.5) | 0.37 | -0.51 | 1.25 | 0.17 |
| **9.5 years** |  |  |  |  |  |  |  |  |  |  |
| Age (yr) | 414 | 9.35 | (0.11) | 48 | 9.40 | (0.10) | -0.05 | -0.10 | 0.01 | 0.004 |
| Height (cm) | 414 | 130.7 | (5.7) | 48 | 131.2 | (5.9) | -0.44 | -3.34 | 2.46 | 0.6 |
| Sum of skinfold thickness (mm) (mm)(mm)* | 414 | 17.7 | (6.2) | 48 | 16.9 | (7.1) | 1.30 | -1.77 | 4.36 | 0.2 |
| Weight (kg) | 414 | 25.0 | (4.4) | 48 | 24.7 | (4.4) | 0.34 | -1.88 | 2.56 | 0.6 |
| **13.5 years** |  |  |  |  |  |  |  |  |  |  |
| Age (yr) | 414 | 13.51 | (0.14) | 72 | 13.52 | (0.16) | 0.01 | -0.05 | 0.07 | 0.6 |
| Height (cm) | 414 | 153.7 | (6.8) | 72 | 153.9 | (7.8) | -0.60 | -3.35 | 2.14 | 0.5 |
| Sum of skinfold thickness (mm) | 414 | 26.5 | (12.1) | 70 | 26.6 | (13.0) | -0.33 | -5.16 | 4.50 | 0.8 |
| Weight (kg) | 414 | 41.7 | (8.4) | 70 | 41.8 | (9.8) | -0.91 | -4.18 | 2.35 | 0.4 |
| Body Fat (%) | 414 | 21.4 | (7.5) | 71 | 22.2 | (7.6) | -0.81 | -3.51 | 1.90 | 0.3 |
| Systolic BP (mmHg) | 414 | 109.2 | (8.3) | 72 | 109.5 | (7.3) | -0.84 | -4.24 | 2.56 | 0.4 |
| Diastolic BP (mmHg) | 414 | 61.2 | (7.0) | 72 | 60.9 | (6.5) | -0.27 | -3.21 | 2.67 | 0.8 |
| Glucose 0 (mmol/l) | 409 | 5.0 | (0.5) | 68 | 4.9 | (0.4) | 0.08 | -0.15 | 0.31 | 0.2 |
| Insulin 0 (pmol/l) ^a^ | 409 | 39.3 | (27.6,51.9) | 68 | 39.6 | (29.4,55.5) | 0.01 | -0.22 | 0.24 | 0.9 |
| HOMA-IR  ^a^ | 409 | 1.5 | (1.0,1.9) | 68 | 1.4 | (1.1,2.2) | 0.02 | -0.22 | 0.26 | 0.7 |
| Total cholesterol (mmol/l) | 409 | 3.5 | (0.7) | 68 | 3.4 | (0.7) | 0.04 | -0.26 | 0.35 | 0.6 |
| Triglycerides (mmol/l) | 409 | 0.8 | (0.4) | 68 | 0.8 | (0.4) | 0.02 | -0.16 | 0.20 | 0.7 |
| HDL-Cholesterol (mmol/l) | 409 | 1.06 | (0.2) | 68 | 1.05 | (0.3) | 0.01 | -0.10 | 0.12 | 0.8 |
| Mean cognitive score | 414 | 44.5 | (7.7) | 72 | 42.7 | (7.6) | 1.65 | -1.59 | 4.90 | 0.1 |
|  |  |  |  |  |  |  |  |  |  |  |

^a^ Median (Inter Quartile Range).

β (99.9% CI) values represent the difference (included-excluded) between children who were included and not included for the conditional analysis; values derived using linear regression analysis, adjusted for age, sex, and (for 13.5 year outcomes), socio-economic status and pubertal stage.

For gestational age and age variables: values adjusted for sex.

SD: standard deviation; CI: confidence interval; BP: Blood pressure; HOMA-IR: Homeostasis Model Assessment insulin resistance; Sum of skinfold thickness: Sum of triceps and subscapular skinfold thickness
