# Supplementary material for: Linear Growth and Fat and Lean Tissue Gain during Childhood: Associations with Cardiometabolic and Cognitive Outcomes in Adolescent Indian Children
Source: PLoS One. 2015 Nov 17;10(11):e0143231. doi: 10.1371/journal.pone.0143231 (PMC4648488; doi:10.1371/journal.pone.0143231)
Supplement: S2 Table — β represents SD change in outcome variables per SD change in growth over that expected at a given age. Analyses adjusted for age, sex, socio-economic status and pubertal stage. SD: standard deviation, CI: confidence interval; Sum of skinfold thickness: sum of triceps and subscapular skinfold thickness. (DOC) [file pone.0143231.s003.doc]

| **Outcomes**  **at age 13.5 y**  **(SD score)** | **Age** |  | **Conditional measure (SD score)** | | | | | | | | | | | | | |
| --- | --- | --- | --- | --- | --- | --- | --- | --- | --- | --- | --- | --- | --- | --- | --- | --- |
|  |  |  | **Linear growth** | | | |  | **Fat gain** | | | |  | **Lean tissue gain** | | | |
|  |  |  | **β** | **(99.9% CI)** | | **P** |  | **β** | **(99.9% CI)** | | P |  | **β** | **(99.9% CI)** | | P |
| **Height** | Birth |  | 0.29 | 0.20 | 0.38 | <0.001 |  | -0.09 | -0.18 | -0.00 | <0.001 |  | 0.04 | -0.04 | 0.13 | 0.1 |
|  | 0-1y |  | 0.48 | 0.40 | 0.57 | <0.001 |  | -0.05 | -0.14 | 0.03 | 0.05 |  | 0.06 | -0.02 | 0.14 | 0.02 |
|  | 1-2y |  | 0.32 | 0.23 | 0.40 | <0.001 |  | -0.01 | -0.10 | 0.07 | 0.6 |  | 0.05 | -0.04 | 0.13 | 0.1 |
|  | 2-5y |  | 0.34 | 0.26 | 0.43 | <0.001 |  | -0.09 | -0.17 | 0.00 | 0.001 |  | 0.03 | -0.06 | 0.11 | 0.3 |
|  | 5-9.5y |  | 0.34 | 0.25 | 0.43 | <0.001 |  | 0.02 | -0.06 | 0.11 | 0.4 |  | -0.03 | -0.12 | 0.06 | 0.2 |
| **Body mass index** | Birth |  | 0.12 | 0.03 | 0.21 | <0.001 |  | 0.08 | -0.01 | 0.17 | 0.01 |  | 0.10 | 0.01 | 0.18 | <0.001 |
|  | 0-1y |  | 0.01 | -0.08 | 0.10 | 0.8 |  | 0.15 | 0.06 | 0.24 | <0.001 |  | 0.22 | 0.13 | 0.30 | <0.001 |
|  | 1-2y |  | 0.01 | -0.08 | 0.10 | 0.7 |  | 0.07 | -0.03 | 0.16 | 0.02 |  | 0.23 | 0.14 | 0.32 | <0.001 |
|  | 2-5y |  | 0.07 | -0.02 | 0.16 | 0.01 |  | 0.16 | 0.07 | 0.25 | <0.001 |  | 0.36 | 0.27 | 0.44 | <0.001 |
|  | 5-9.5y |  | 0.12 | 0.03 | 0.21 | <0.001 |  | 0.49 | 0.40 | 0.58 | <0.001 |  | 0.30 | 0.21 | 0.39 | <0.001 |
| **Waist-hip ratio** | Birth |  | 0.03 | -0.13 | 0.19 | 0.6 |  | 0.04 | -0.12 | 0.20 | 0.4 |  | -0.03 | -0.19 | 0.12 | 0.5 |
|  | 0-1y |  | -0.10 | -0.25 | 0.06 | 0.03 |  | 0.07 | -0.08 | 0.23 | 0.1 |  | 0.05 | -0.10 | 0.20 | 0.3 |
|  | 1-2y |  | -0.09 | -0.24 | 0.07 | 0.1 |  | 0.07 | -0.08 | 0.23 | 0.1 |  | 0.02 | -0.14 | 0.18 | 0.6 |
|  | 2-5y |  | -0.05 | -0.21 | 0.12 | 0.4 |  | 0.16 | 0.01 | 0.32 | <0.001 |  | 0.12 | -0.04 | 0.27 | 0.01 |
|  | 5-9.5y |  | -0.01 | -0.16 | 0.15 | 0.9 |  | 0.31 | 0.15 | 0.46 | <0.001 |  | 0.01 | -0.15 | 0.17 | 0.8 |
| **Sum of skinfold thickness** | Birth |  | 0.11 | 0.00 | 0.23 | <0.001 |  | 0.14 | 0.02 | 0.25 | <0.001 |  | 0.03 | -0.08 | 0.14 | 0.3 |
|  | 0-1y |  | 0.09 | -0.02 | 0.20 | 0.01 |  | 0.22 | 0.12 | 0.33 | <0.001 |  | 0.06 | -0.05 | 0.17 | 0.1 |
|  | 1-2y |  | 0.08 | -0.03 | 0.19 | 0.02 |  | 0.19 | 0.07 | 0.30 | <0.001 |  | 0.11 | -0.00 | 0.22 | 0.001 |
|  | 2-5y |  | 0.18 | 0.06 | 0.29 | <0.001 |  | 0.22 | 0.11 | 0.33 | <0.001 |  | 0.15 | 0.04 | 0.26 | <0.001 |
|  | 5-9.5y |  | 0.14 | 0.03 | 0.25 | <0.001 |  | 0.56 | 0.45 | 0.67 | <0.001 |  | 0.11 | -0.01 | 0.22 | 0.002 |
| **Fat%** | Birth |  | 0.07 | -0.08 | 0.21 | 0.1 |  | 0.05 | -0.09 | 0.20 | 0.2 |  | -0.01 | -0.16 | 0.13 | 0.7 |
|  | 0-1y |  | 0.06 | -0.08 | 0.20 | 0.2 |  | 0.10 | -0.05 | 0.24 | 0.03 |  | 0.08 | -0.06 | 0.22 | 0.1 |
|  | 1-2y |  | 0.03 | -0.11 | 0.18 | 0.4 |  | 0.11 | -0.04 | 0.25 | 0.02 |  | 0.12 | -0.03 | 0.26 | 0.01 |
|  | 2-5y |  | 0.09 | -0.06 | 0.23 | 0.1 |  | 0.16 | 0.02 | 0.30 | <0.001 |  | 0.11 | -0.03 | 0.26 | 0.01 |
|  | 5-9.5y |  | 0.09 | -0.06 | 0.23 | 0.04 |  | 0.44 | 0.29 | 0.58 | <0.001 |  | 0.11 | -0.04 | 0.26 | 0.01 |

**S2 Table. Regression Coefficients for Associations Between Conditional Growth Variables and Anthropometry and Body Composition at 13.5 Years.**

β represents SD change in outcome variables per SD change in growth over that expected at a given age. Analyses adjusted for age, sex, socio-economic status and pubertal stage**.**

SD: standard deviation; CI: confidence interval; Sum of skinfold thickness: sum of triceps and subscapular skinfold thickness.
