# Supplementary material for: Linear Growth and Fat and Lean Tissue Gain during Childhood: Associations with Cardiometabolic and Cognitive Outcomes in Adolescent Indian Children
Source: PLoS One. 2015 Nov 17;10(11):e0143231. doi: 10.1371/journal.pone.0143231 (PMC4648488; doi:10.1371/journal.pone.0143231)
Supplement: S4 Table — β represents SD change in outcome variables per SD change in growth over that expected at a given age. Analyses adjusted for age, sex, socio-economic status and pubertal stage. SD: standard deviation, CI: confidence interval. (DOC) [file pone.0143231.s005.doc]

**S4 Table. Regression Coefficients for Associations Between Conditional Growth Variables and Cognitive Outcomes at 13.5 years.**

| **Outcomes**  **at age 13.5 y**  **(SD score)** | **Age** |  | **Conditional measure (SD score)** | | | | | | | | | | | | | |
| --- | --- | --- | --- | --- | --- | --- | --- | --- | --- | --- | --- | --- | --- | --- | --- | --- |
|  |  |  | **Linear growth** | | | |  | **Fat gain** | | | |  | **Lean tissue gain** | | | |
|  |  |  | **β** | **(99.9% CI)** | | **P** |  | **β** | **(99.9% CI)** | | **P** |  | **β** | **(99.9% CI)** | | **P** |
|  |  |  |  |  |  |  |  |  |  |  |  |  |  |  |  |  |
| **Visuo-spatial ability**  **(Koh’s block design)** | Birth |  | 0.03 | -0.15 | 0.20 | 0.6 |  | 0.02 | -0.15 | 0.19 | 0.7 |  | 0.14 | -0.03 | 0.31 | 0.01 |
|  | 0-1y |  | 0.12 | -0.05 | 0.28 | 0.02 |  | -0.00 | -0.17 | 0.16 | 0.9 |  | 0.03 | -0.13 | 0.20 | 0.5 |
|  | 1-2y |  | 0.03 | -0.14 | 0.20 | 0.6 |  | 0.03 | -0.14 | 0.20 | 0.6 |  | 0.01 | -0.16 | 0.18 | 0.9 |
|  | 2-5y |  | -0.01 | -0.18 | 0.16 | 0.8 |  | -0.02 | -0.18 | 0.15 | 0.8 |  | 0.04 | -0.12 | 0.21 | 0.4 |
|  | 5-9.5y |  | 0.08 | -0.08 | 0.25 | 0.1 |  | 0.06 | -0.11 | 0.23 | 0.2 |  | -0.03 | -0.20 | 0.14 | 0.5 |
|  | 9.5-13.5y |  | 0.10 | -0.07 | 0.28 | 0.1 |  | -0.06 | -0.23 | 0.11 | 0.2 |  | -0.05 | -0.22 | 0.12 | 0.3 |
|  |  |  |  |  |  |  |  |  |  |  |  |  |  |  |  |  |
| **Language production**  **(Verbal fluency)** | Birth |  | 0.12 | -0.06 | 0.29 | 0.03 |  | -0.04 | -0.21 | 0.13 | 0.4 |  | 0.00 | -0.17 | 0.17 | 0.9 |
|  | 0-1y |  | 0.08 | -0.10 | 0.25 | 0.1 |  | 0.03 | -0.14 | 0.20 | 0.6 |  | 0.06 | -0.11 | 0.22 | 0.3 |
|  | 1-2y |  | 0.08 | -0.10 | 0.25 | 0.1 |  | 0.05 | -0.13 | 0.22 | 0.4 |  | 0.06 | -0.12 | 0.23 | 0.3 |
|  | 2-5y |  | 0.02 | -0.16 | 0.20 | 0.7 |  | -0.06 | -0.23 | 0.11 | 0.3 |  | 0.08 | -0.09 | 0.25 | 0.1 |
|  | 5-9.5y |  | 0.03 | -0.14 | 0.20 | 0.6 |  | 0.05 | -0.13 | 0.22 | 0.4 |  | 0.08 | -0.09 | 0.26 | 0.1 |
|  | 9.5-13.5y |  | -0.01 | -0.19 | 0.17 | 0.8 |  | -0.05 | -0.22 | 0.13 | 0.4 |  | 0.05 | -0.13 | 0.22 | 0.4 |
|  |  |  |  |  |  |  |  |  |  |  |  |  |  |  |  |  |
| **Long-term memory and retrieval**  **(Atlantis)** | Birth |  | -0.03 | -0.20 | 0.14 | 0.6 |  | 0.09 | -0.08 | 0.26 | 0.1 |  | 0.06 | -0.11 | 0.23 | 0.2 |
|  | 0-1y |  | 0.08 | -0.09 | 0.25 | 0.1 |  | -0.00 | -0.17 | 0.16 | 1.0 |  | 0.08 | -0.08 | 0.24 | 0.1 |
|  | 1-2y |  | 0.02 | -0.15 | 0.19 | 0.7 |  | 0.03 | -0.14 | 0.19 | 0.6 |  | -0.01 | -0.18 | 0.16 | 0.8 |
|  | 2-5y |  | 0.01 | -0.17 | 0.18 | 0.9 |  | -0.05 | -0.22 | 0.12 | 0.3 |  | 0.07 | -0.10 | 0.24 | 0.2 |
|  | 5-9.5y |  | -0.09 | -0.26 | 0.08 | 0.1 |  | 0.10 | -0.07 | 0.27 | 0.1 |  | -0.02 | -0.19 | 0.15 | 0.7 |
|  | 9.5-13.5y |  | 0.02 | -0.16 | 0.20 | 0.7 |  | 0.02 | -0.15 | 0.19 | 0.7 |  | -0.01 | -0.17 | 0.16 | 0.9 |
|  |  |  |  |  |  |  |  |  |  |  |  |  |  |  |  |  |
| **Reasoning**  **(Pattern reasoning)** | Birth |  | 0.08 | -0.10 | 0.25 | 0.1 |  | 0.01 | -0.16 | 0.18 | 0.8 |  | 0.07 | -0.10 | 0.23 | 0.2 |
|  | 0-1y |  | 0.10 | -0.07 | 0.26 | 0.1 |  | -0.02 | -0.18 | 0.15 | 0.7 |  | 0.04 | -0.12 | 0.20 | 0.4 |
|  | 1-2y |  | 0.08 | -0.09 | 0.25 | 0.1 |  | -0.02 | -0.19 | 0.15 | 0.6 |  | 0.04 | -0.13 | 0.21 | 0.5 |
|  | 2-5y |  | 0.01 | -0.16 | 0.18 | 0.9 |  | -0.06 | -0.23 | 0.10 | 0.2 |  | 0.09 | -0.08 | 0.25 | 0.1 |
|  | 5-9.5y |  | 0.07 | -0.10 | 0.24 | 0.2 |  | 0.06 | -0.11 | 0.23 | 0.2 |  | 0.05 | -0.12 | 0.22 | 0.3 |
|  | 9.5-13.5y |  | 0.02 | -0.16 | 0.20 | 0.7 |  | 0.01 | -0.16 | 0.18 | 0.9 |  | 0.04 | -0.13 | 0.21 | 0.4 |
|  |  |  |  |  |  |  |  |  |  |  |  |  |  |  |  |  |
| **Short-term memory (Word order)** | Birth |  | 0.07 | -0.10 | 0.25 | 0.2 |  | -0.05 | -0.22 | 0.12 | 0.4 |  | 0.10 | -0.06 | 0.27 | 0.04 |
|  | 0-1y |  | 0.01 | -0.15 | 0.18 | 0.8 |  | 0.03 | -0.13 | 0.20 | 0.5 |  | 0.09 | -0.07 | 0.25 | 0.1 |
|  | 1-2y |  | -0.01 | -0.18 | 0.16 | 0.8 |  | 0.00 | -0.17 | 0.17 | 1.0 |  | 0.02 | -0.15 | 0.19 | 0.7 |
|  | 2-5y |  | 0.00 | -0.17 | 0.18 | 0.9 |  | -0.09 | -0.26 | 0.08 | 0.1 |  | 0.07 | -0.10 | 0.23 | 0.2 |
|  | 5-9.5y |  | 0.03 | -0.14 | 0.20 | 0.6 |  | 0.01 | -0.16 | 0.18 | 0.8 |  | 0.01 | -0.16 | 0.18 | 0.9 |
|  | 9.5-13.5y |  | -0.02 | -0.19 | 0.16 | 0.8 |  | -0.06 | -0.23 | 0.11 | 0.3 |  | -0.04 | -0.21 | 0.13 | 0.4 |
|  |  |  |  |  |  |  |  |  |  |  |  |  |  |  |  |  |
| **Short-term memory (Coding)** | Birth |  | 0.04 | -0.13 | 0.22 | 0.4 |  | 0.07 | -0.10 | 0.24 | 0.2 |  | -0.02 | -0.19 | 0.15 | 0.7 |
|  | 0-1y |  | 0.14 | -0.03 | 0.31 | 0.01 |  | 0.05 | -0.11 | 0.22 | 0.3 |  | 0.04 | -0.12 | 0.21 | 0.4 |
|  | 1-2y |  | 0.07 | -0.10 | 0.24 | 0.2 |  | -0.00 | -0.17 | 0.17 | 1.0 |  | 0.05 | -0.12 | 0.22 | 0.3 |
|  | 2-5y |  | 0.08 | -0.09 | 0.26 | 0.1 |  | -0.07 | -0.24 | 0.09 | 0.1 |  | 0.13 | -0.04 | 0.30 | 0.01 |
|  | 5-9.5y |  | 0.07 | -0.10 | 0.24 | 0.2 |  | 0.07 | -0.11 | 0.24 | 0.2 |  | 0.04 | -0.14 | 0.21 | 0.5 |
|  | 9.5-13.5y |  | -0.00 | -0.18 | 0.18 | 0.9 |  | -0.07 | -0.24 | 0.10 | 0.2 |  | 0.01 | -0.17 | 0.18 | 0.9 |
|  |  |  |  |  |  |  |  |  |  |  |  |  |  |  |  |  |
| **Mean cognitive score** | Birth |  | 0.03 | -0.14 | 0.20 | 0.5 |  | 0.05 | -0.12 | 0.21 | 0.4 |  | 0.11 | -0.05 | 0.27 | 0.02 |
|  | 0-1y |  | 0.15 | -0.01 | 0.32 | 0.0 |  | 0.01 | -0.15 | 0.17 | 0.9 |  | 0.08 | -0.08 | 0.24 | 0.1 |
|  | 1-2y |  | 0.06 | -0.10 | 0.23 | 0.2 |  | 0.02 | -0.14 | 0.18 | 0.7 |  | 0.02 | -0.14 | 0.18 | 0.7 |
|  | 2-5y |  | 0.02 | -0.15 | 0.19 | 0.7 |  | -0.06 | -0.22 | 0.10 | 0.2 |  | 0.11 | -0.06 | 0.27 | 0.03 |
|  | 5-9.5y |  | 0.05 | -0.11 | 0.22 | 0.3 |  | 0.09 | -0.07 | 0.25 | 0.1 |  | 0.00 | -0.17 | 0.16 | 0.9 |
|  | 9.5-13.5y |  | 0.06 | -0.11 | 0.23 | 0.3 |  | -0.05 | -0.22 | 0.12 | 0.3 |  | -0.01 | -0.17 | 0.16 | 0.9 |

β represents SD change in outcome variables per SD change in growth over that expected at a given age. Analyses adjusted for age, sex, socio-economic status and pubertal stage**.**

SD: standard deviation; CI: confidence interval.
